# Recent Advances in Metal–Organic Framework-Based Nanozymes for Intelligent Microbial Biosensing: A Comprehensive Review of Biomedical and Environmental Applications

**DOI:** 10.3390/bios15070437

**Published:** 2025-07-07

**Authors:** Alemayehu Kidanemariam, Sungbo Cho

**Affiliations:** 1 Department of Electronic Engineering, Gachon University, Seongnam-si 13120, Republic of Korea; alemayehukim@gachon.ac.kr; 2 Department of Semiconductor Engineering, Gachon University, Seongnam-si 13120, Republic of Korea; 3 Gachon Advanced Institute for Health Science & Technology, Gachon University, Incheon 21999, Republic of Korea

**Keywords:** metal–organic framework-based nanozymes, microbial biosensing, point-of-care diagnostics, enzyme mimetics, biomedical, environmental

## Abstract

Metal–organic framework (MOF)-based nanozymes represent a groundbreaking frontier in advanced microbial biosensing, offering unparalleled catalytic precision and structural tunability to mimic natural enzymes with superior stability and specificity. By engineering the structural features and forming composites, MOFs are precisely tailored to amplify nanozymatic activity, enabling the highly sensitive, rapid, and cost-effective detection of a broad spectrum of microbial pathogens critical to biomedical diagnostics and environmental monitoring. These advanced biosensors surpass traditional enzyme systems in robustness and reusability, integrating seamlessly with smart diagnostic platforms for real-time, on-site microbial identification. This review highlights cutting-edge developments in MOF nanozyme design, composite engineering, and signal transduction integration while addressing pivotal challenges such as biocompatibility, complex matrix interference, and scalable manufacturing. Looking ahead, the convergence of multifunctional MOF nanozymes with portable technologies and optimized in vivo performance will drive transformative breakthroughs in early disease detection, antimicrobial resistance surveillance, and environmental pathogen control, establishing a new paradigm in next-generation smart biosensing.

## 1. Introduction

The global burden of infectious diseases, increase in antimicrobial resistance, and increasing environmental contamination have underscored the urgent need for rapid, sensitive, and reliable microbial detection technologies [1,2,3]. The early identification of pathogenic microorganisms is essential for effective outbreak response, informed treatment decisions, and public health management in settings ranging from hospitals and food production to water quality monitoring [4,5].

Traditional microbial detection methods including culture-based assays, polymerase chain reaction (PCR), and enzyme-linked immunosorbent assays (ELISA) have long served as diagnostic gold standards [6,7,8]. However, these approaches are often time-intensive, laborious, and dependent on sophisticated instrumentation and trained personnel [9]. These constraints limit their applicability in decentralized and resource-limited environments where rapid decision making is critical [10,11].

To overcome these limitations, significant research efforts have been directed toward the development of advanced biosensors using functional nanomaterials [12,13]. Nanomaterials such as noble metal nanoparticles (e.g., Au, Ag), carbon-based nanostructures (e.g., graphene oxide, carbon nanotubes), quantum dots, and magnetic nanoparticles have been explored for their unique physicochemical properties, enabling diverse detection modalities such as fluorescence, surface-enhanced Raman scattering (SERS), and electrochemical sensing [14,15,16]. These systems provide high sensitivity and rapid response but often face practical challenges, including poor stability under physiological conditions, limited biorecognition specificity, and potential cytotoxicity [17,18].

Enzyme-based biosensors offer high catalytic efficiency and specificity but suffer from inherent limitations such as environmental instability, high cost, and short shelf life due to protein denaturation [19,20,21]. These drawbacks have driven the emergence of nanozymes, which are nanomaterials that mimic natural enzymatic activity while offering enhanced robustness and functional versatility [22,23]. Among various nanozyme candidates, metal–organic frameworks (MOFs) have garnered increasing attention for microbial biosensing due to their highly tunable structures and multifunctionality [24,25].

MOFs are crystalline, porous materials composed of metal nodes coordinated to organic linkers, forming extended three-dimensional architectures [26]. Their ultrahigh surface area, tunable pore environment, and modular chemistry make MOFs ideal platforms for mimicking enzymatic active sites and incorporating biorecognition elements [27,28]. Unlike conventional nanomaterials, MOFs offer the advantage of structural programmability at the molecular level, allowing for precise control over catalytic behavior, target specificity, and sensor performance [29].

In the context of microbial detection, MOFs can serve dual roles: as nanozyme catalysts facilitating signal generation and as scaffolds for immobilizing recognition units such as aptamers, antibodies, DNA probes, and molecularly imprinted polymers [30,31]. These attributes have enabled the creation of highly sensitive and selective biosensors capable of detecting a wide range of microbial targets including bacteria (e.g., *Escherichia coli*, *Salmonella*, *Staphylococcus aureus*) and viruses (e.g., influenza, SARS-CoV-2) through various transduction mechanisms such as colorimetric, fluorescent, electrochemical, and luminescent readouts [32,33,34].

Furthermore, the adaptability of MOFs has supported their integration into portable and digital diagnostic platforms [35]. The combination of MOF-based nanozymes with smartphone interfaces, paper-based sensors, and microfluidic systems is paving the way for next-generation point-of-care (POC) diagnostic tools [36]. These compact and affordable systems hold promise for on-site microbial monitoring in clinical, agricultural, and environmental applications, enabling real-time decision making and outbreak containment [25,37].

This review aims to provide a comprehensive overview of the recent progress in MOF-based nanozymes for microbial detection and assay development. We examine key aspects of MOF nanozyme design, including catalytic mechanism tuning, surface modification strategies, and hybrid material construction. Emphasis is placed on the integration of MOFs with biorecognition molecules to enhance detection specificity and performance.

In addition, we critically analyze the current challenges associated with MOF nanozyme-based biosensors, such as matrix interference in complex samples, reproducibility issues, biocompatibility, and long-term stability. Potential strategies to address these challenges such as advanced composite engineering, robust synthesis protocols, and biointerface optimization are discussed. Finally, we explore future directions for the field, including the incorporation of MOFs into Internet of Things (IoT)-enabled smart diagnostic networks and regulatory considerations for their clinical translation.

By synthesizing insights from materials science, microbiology, and sensor engineering, this review offers a strategic roadmap for harnessing MOFs to develop next-generation biosensing technologies for microbial detection.

## 2. Fundamentals of MOF Nanozymes

The integration of MOFs with nanozyme technologies has opened new avenues for the development of next-generation biosensors [38]. MOF nanozymes synergistically combine the advantages of MOFs, such as high porosity, structural tunability, and chemical functionality, with the catalytic properties of nanozymes that mimic natural enzymes [39].

### 2.1. MOFs: Structure and Properties

**Scheme 1:**
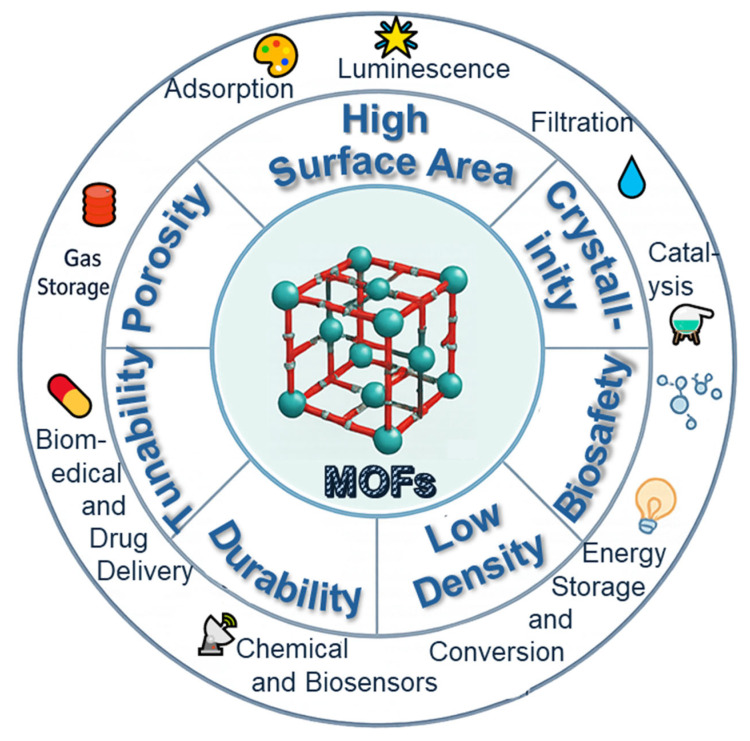
Schematic overview of MOF properties and their application areas. MOF, metal–organic framework.

MOFs are crystalline porous materials composed of metal ions or clusters coordinated with organic ligands that form highly ordered network structures [40]. Their defining characteristics include ultrahigh surface areas, tunable pore sizes, and the ability to be chemically tailored at both the metal center and linker levels [41]. This structural tunability allows for the precise modulation of surface chemistry and functionality, enabling MOFs to be customized for a wide range of application areas such as catalysis, gas storage, drug delivery, and biosensing (Scheme 1) [42,43,44]. In the context of biosensing, their porosity and chemical versatility enable the selective adsorption and interaction with target microbial analytes, laying the structural foundation for nanozyme integration.

### 2.2. Nanozymes: Definition and Classification

Nanozymes are nanomaterials that exhibit intrinsic enzyme-like catalytic activities, mimicking natural enzymes such as peroxidase, oxidase, catalase, and superoxide dismutase [45,46]. Unlike natural enzymes, nanozymes offer significant advantages, including higher operational stability, ease of large-scale production, and enhanced resistance to harsh conditions, broadening their practical applications [47]. These properties enable nanozymes to serve diverse roles in biosensing, therapeutics, and environmental monitoring. Nanozymes can be broadly classified based on their catalytic mechanisms and material composition into three main categories: metal-based, carbon-based, and composite nanozymes [48]. Metal-based nanozymes typically consist of metal or metal oxide nanoparticles (e.g., Fe, Ce, Mn oxides) and primarily mimic oxidoreductase enzymes. Carbon-based nanozymes involve functionalized carbon materials like graphene and carbon nanotubes, providing stable and tunable enzyme-like activities [49]. Composite nanozymes combine metals, metal oxides, and polymers to create multifunctional catalysts with enhanced versatility [50].

Representative examples illustrate these categories and their associated applications. FePPOP-1, a porphyrin-based porous polymer synthesized via Sonogashira–Hagihara coupling, exhibits peroxidase-like activity by catalyzing the oxidation of 3,3′,5,5′-tetramethylbenzidine (TMB) in the presence of hydrogen peroxide, enabling the sensitive colorimetric detection of H_2_O_2_, glucose, and various antioxidants [51]. This example highlights how metal-based nanozymes can amplify biosensing signals through their catalytic properties. In composite nanozymes, Smutok et al. developed a biosensor combining carbon microfiber-based peroxidase-mimetic nanozymes functionalized with hemin and decorated with platinum nanoparticles along with microbial lactate oxidase, achieving selective and sensitive L-lactate detection relevant for food quality control and clinical diagnostics [52]. This synergy between nanozymes and biological enzymes improves selectivity and sensitivity.

**Figure 1:**
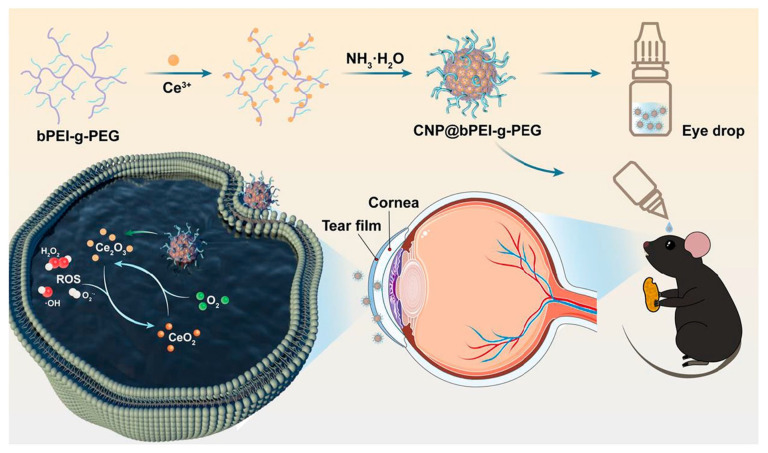
Schematic representation of CNP@bPEI-g-PEG nanozymes and their antioxidant mechanism in alleviating oxidative damage associated with dry eye disease (DED). Copyright 2022, Regenerative Biomaterials [53].

In therapeutic applications, cerium oxide nanozymes modified with branched poly(ethylene imine)-graft-poly(ethylene glycol) (CNP@bPEI-g-PEG) mimic both superoxide dismutase and catalase activities, enabling regenerative reactive oxygen species (ROS) scavenging through cerium redox cycling. In vivo experiments confirmed their therapeutic efficacy in a dry eye disease model, demonstrating reduced oxidative damage, the restoration of corneal and conjunctival integrity, and increased goblet cell density [53]. Figure 1 schematically illustrates this therapeutic mechanism, showing how CNP@bPEI-g-PEG nanozymes mimic enzymatic activities, scavenge ROS via redox cycling between Ce^3+^ and Ce^4+^, and exert protective effects on ocular tissues. This work underscores the clinical potential of metal oxide nanozymes in treating oxidative stress-related disorders.

Finally, multifunctional composite nanozymes like Mn_3_O_4_@g-C_3_N_4_ exhibit diverse enzyme-like activities including superoxide dismutase, catalase, oxidase, and peroxidase allowing efficient ROS scavenging and serving as label-free colorimetric probes for phenolic pollutant detection, thus bridging environmental remediation and sensing applications [54]. Collectively, these studies demonstrate that understanding nanozyme classification based on catalytic mechanism and material composition facilitates the rational design of nanozymes with tailored properties for diverse and impactful applications, highlighting their potential as robust and versatile alternatives to natural enzymes.

### 2.3. MOFs as Nanozyme Platforms

MOFs provide a unique and versatile scaffold for constructing nanozymes, owing to their ability to spatially isolate catalytic sites, host metal centers with redox activity, and allow for the diffusion of substrates through their porous matrices [55]. The confinement effect and high local concentration of active sites within MOFs often lead to enhanced catalytic efficiency compared with other nanozyme platforms [56]. Moreover, the modularity of MOF design enables the rational incorporation of functional groups and metal species tailored to specific microbial sensing mechanisms [57]. As such, MOFs serve not only as passive supports but also as active participants in enzyme-mimetic catalysis, offering vast potential in intelligent biosensing applications [58]. A summary of the key concepts discussed in this section is provided in Table 1.

## 3. Design Strategies for MOF Nanozymes in Microbial Biosensing

The performance of MOF nanozymes in microbial biosensing applications is strongly influenced by how their structural and functional properties are engineered to interact effectively with microbial targets such as bacterial cells or their biomarkers [59]. Strategic modifications at the metal center, organic linker, particle size, and hybrid composition levels enable precise control over catalytic activity, selectivity, and substrate recognition, all of which are critical for detecting microorganisms or their associated molecules in complex environments [44,60].

### 3.1. Metal Center Engineering

The metal nodes within an MOF play a pivotal role in defining the catalytic behavior of nanozymes [61]. By selecting metals such as Fe, Co, Cu, or Mn with redox activity, researchers can tailor enzyme-mimicking functions like peroxidase-like or oxidase-like activity [62]. Such tailoring enhances the nanozyme’s ability to detect microbial entities by catalyzing colorimetric, fluorescent, or electrochemical signals in response to bacterial presence or activity [63].

One highly relevant study engineered dual atomic site catalysts (DASCs) within an MOF-808 framework to detect methicillin-resistant *Staphylococcus aureus* (MRSA). Through formic acid modulation, abundant defect sites were introduced to facilitate Co atom incorporation, creating Co_2_–O_10_ dual sites with high metal loading (11.1 wt%). These DASCs exhibited exceptional peroxidase-like activity, amplifying chemiluminescence by ~5800-fold in a luminol-H_2_O_2_ system. They enabled ultrasensitive MRSA detection over a range of 10^2^−10^7^ CFU/mL with a limit of detection (LOD) of 47 CFU/mL and also proved effective in antibiotic susceptibility testing [64]. This underscores how precise metal center engineering can directly support real-time microbial diagnostics.

Another example involves a gold nanoparticle (AuNP)-doped bimetallic CuZr-MOF nanozyme, used for the electrochemical detection of *Salmonella* Typhimurium. Functionalized with signal DNA probes, the platform achieved high sensitivity (LOD = 0.82 CFU/mL) via the peroxidase-mimetic electrocatalysis of H_2_O_2_ and was successfully applied to quantify *Salmonella* DNA in milk [65]. Here, the synergy among Cu(II), Zr(IV), and AuNPs significantly enhanced microbial target detection.

To address microbial endotoxin detection, Cu^2+^-modified nanoscale MOFs (Cu^2+^-NMOFs) were developed as electrochemical biosensors for lipopolysaccharide (LPS), a key bacterial component. Cu^2+^-NMOFs served as both recognition elements (via electrostatic binding to LPS carbohydrate moieties) and nanozymes catalyzing dopamine oxidation into aminochrome. The system demonstrated ultra-low LOD (0.61 pg/mL) and broad linearity (0.0015–750 ng/mL) in serum samples, offering superior specificity compared to traditional LPS-binding proteins or aptamers [66].

A direct fluorescent biosensing approach was established using a turn-on Fe-MOF platform to detect two major foodborne pathogens, *Escherichia coli* and *Staphylococcus aureus*. Fluorescence enhancement occurred due to surface interactions with bacterial membranes, modulating electron transfer. The biosensor achieved LODs of 0.464 log CFU/mL (*Staphylococcus aureus*) and 0.584 log CFU/mL (*Escherichia coli*) in PBS, water, and milk, all under 1 h [67]. This highlights the practical and rapid potential of Fe-MOF nanozymes in food safety-related microbial diagnostics.

Additionally, Shen et al. synthesized an amine-functionalized bimetallic Fe–Ni MOF-74 nanozyme for detecting pathogenic *Staphylococcus aureus*. Through a layered double hydroxide-assisted method, Fe and Ni worked synergistically to enhance peroxidase-like activity. The platform showed robust detection performance for both small biomolecules and bacterial pathogens, making it highly suitable for microbial biosensing contexts [68]. These results demonstrate the promise of metal-doped MOF nanozymes as adaptable platforms for microbial biosensing and broader bioanalytical applications.

**Figure 2:**
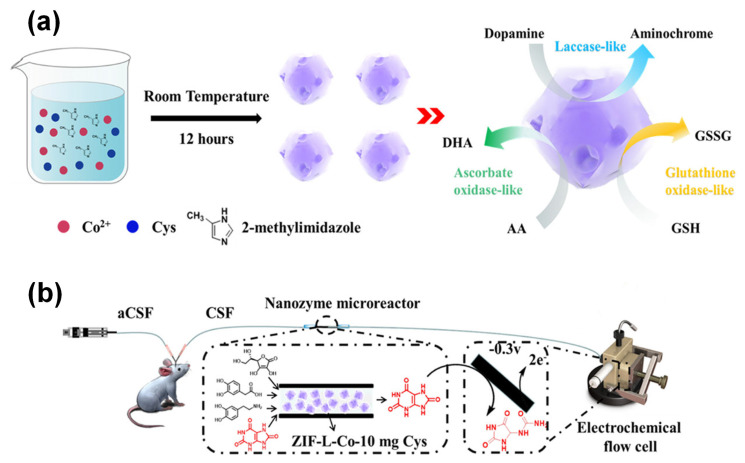
Diagrammatic representation of (**a**) the synthesis process and key characteristics of ZIF-L-Co-10 mg Cys and (**b**) real-time uric acid monitoring using a microreactor containing immobilized ZIF-L-Co-10 mg Cys, integrated with in vivo microdialysis and electrochemical sensing. Copyright 2021 ACS [60].

Defect engineering was applied by Ren et al. to a Co-based MOF (ZIF-L-Co), using cysteine to introduce coordination disruptions that enhanced oxidase-like activity [60]. This approach disrupted the coordination environment between cobalt and nitrogen atoms, leading to lattice distortion and enhanced oxygen adsorption capacity. The resulting nanozyme exhibited significantly improved oxidase-like activities, including over fivefold, twofold, and threefold increases in ascorbate oxidase, glutathione oxidase, and laccase-like activities, respectively (Figure 2). When integrated into an online electrochemical sensing system, the modified MOF effectively eliminated common interferences during uric acid detection in a biological environment. While the study primarily targeted neurological biomarkers, the metal defect engineering and multi-enzyme mimicry strategy hold strong translational potential for microbial biosensing platforms. By tailoring the metal–ligand coordination and catalytic reactivity, such MOFs could be adapted for selective pathogen detection, particularly when coupled with DNA probes or microbial recognition elements.

**Figure 3:**
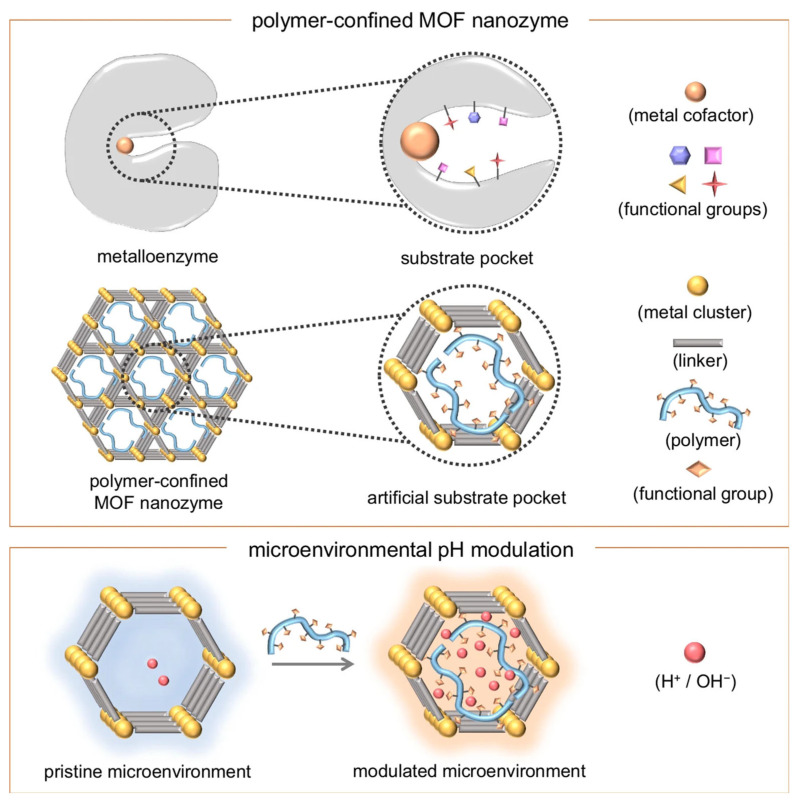
Overcoming pH constraints to enhance the catalytic performance of MOF-based nanozymes. Copyright 2024, Nature Communications [69].

Similarly, controlling the internal microenvironment of MOF nanozymes is another essential design strategy for improving microbial detection. In one strategy, Li et al. embedded poly(acrylic acid) (PAA) into PCN-222-Fe to regulate local pH, boosting peroxidase activity at physiological conditions (pH 7.4), a crucial requirement for stable enzymatic sensing in biological samples [69]. Although originally designed for multi-enzyme cascades, this microenvironment modulation approach is directly translatable to microbial sensing systems operating under physiologic or food-relevant pH ranges (Figure 3).

Therefore, tailoring metal centers within MOFs through doping, nanoparticle incorporation, and microenvironmental control significantly enhances nanozyme catalytic activity and specificity. These improvements enable the sensitive detection of diverse biological targets and facilitate integration into practical diagnostic platforms, underscoring the promise of engineered MOF nanozymes for advanced biomedical applications.

### 3.2. Ligand Functionalization

The chemical modification of organic linkers in MOFs offers a powerful route to enhance microbial biosensing performance by improving molecular recognition, signal amplification, and electron transfer [70]. Functional groups such as aptamers, antibodies, redox-active moieties, or fluorophores can be integrated into the ligand backbone, enabling selective binding to microbial biomarkers and facilitating catalytic signal transduction [71,72]. This tunability is central to achieving high specificity, sensitivity, and robustness in MOF-based biosensing platforms [73].

An illustrative approach involves the use of ultrathin two-dimensional MOFs (2D-MOFs) conjugated with fluorescent dye-labeled peptides, forming a versatile sensor array for microbial fingerprinting [74]. These 2D-MOFs exhibit high surface area and strong fluorescence quenching capability, efficiently adsorbing the labeled peptides and suppressing emissions. Upon pathogen introduction, diverse non-specific interactions such as electrostatic or biomolecular binding induce peptide displacement, resulting in fluorescence recovery. This pattern shift enables rapid and accurate discrimination between microbial species with 100% classification accuracy, even in complex biological matrices like urine, within approximately 15 min.

Ligand functionalization can also impart dual functionality for detection and disinfection. For example, boronic acid-modified UiO-66 (Zr-UiO-66-B(OH)_2_) exploits selective interactions with bacterial glycolipids for label-free fluorescence detection, achieving a detection limit as low as 1.0 CFU/mL [75]. Under light irradiation, the same MOF generates ROS, conferring photocatalytic antibacterial activity. This multifunctional design underscores the potential of rational ligand engineering for integrated biosensing and antimicrobial functions.

Targeted microbial recognition has also been achieved through the conjugation of NH_2_-MIL-101(Fe) with lytic bacteriophages, immobilized via glutaraldehyde cross-linking [76]. The MOF scaffold enhances phage stability and signal amplification, enabling the rapid and specific detection of *Escherichia coli* at levels as low as 652 CFU/mL within 10–12 min. The approach combines pathogen-specific recognition with MOF-mediated signal output, directly addressing needs in real-time microbial diagnostics.

In another ligand-engineered platform, NH_2_-MIL-53(Fe) was conjugated with galactose and mannose to create glycosylated MOFs (Glyco-MOFs) for the selective detection of *Pseudomonas aeruginosa* and *Escherichia coli* [77]. The carbohydrate ligands target lectins on bacterial surfaces, enabling label-free luminescent detection with detection limits of 8 CFU/mL and 202 CFU/mL, respectively. The Glyco-MOFs demonstrated high specificity and environmental stability, suitable for real-world pathogen monitoring.

Innovative ligand strategies can also enable programmable biosensing behavior. For example, ZIF-8 was functionalized and encapsulated within DNA-based surfactant micelles to form nucleic acid nanocapsules (NANs) [78]. This architecture allowed dual-gated enzymatic activity and analyte release controlled by environmental stimuli such as pH and enzymatic degradation. Such ligand–shell coordination provides tunable signal output and enhanced selectivity in biologically complex systems.

Ligand functionalization also enhances electrochemical biosensing performance. Shahrokhian et al. developed an aptamer-based MOF biosensor for *Escherichia coli* O157:H7, using amino-functionalized MOF linkers to stably immobilize aptamers via glutaraldehyde chemistry [79]. The incorporation of conductive polyaniline into the MOF improved electron transfer, resulting in a wide detection range (2.1 × 10^1^ to 2.1 × 10^7^ CFU/mL) and a low detection limit of 2 CFU/mL.

Beyond functional group incorporation, ligand intercalation can stabilize atomically dispersed catalytic sites within MOFs. In a dual MOF-on-MOF system (CoNi-MOF@PCN-224/Fe), ligand intercalation tuned the coordination environment to promote Fenton-like catalysis and efficient electron transfer, leading to a 17-fold enhancement in electrochemiluminescence (ECL) for H_2_O_2_ detection [80]. Similarly, the integration of metalloporphyrin ligands in PMOF(Fe) provided abundant Fe active sites with intrinsic peroxidase-like activity [63]. Anchoring Pt nanoparticles on the ligand interface further amplified catalytic performance via synergistic electron transfer, demonstrating superior biosensing capabilities.

These examples collectively demonstrate that strategic ligand engineering via functionalization, conjugation, or intercalation can substantially elevate MOF nanozyme performance in microbial biosensing by integrating recognition specificity with catalytic amplification.

### 3.3. Morphology and Size Control

The morphology and size of MOFs critically influence their performance in microbial biosensing applications. Nanoscale MOFs with well-defined shapes and dimensions offer enhanced surface reactivity, improved analyte diffusion, and superior interactions with microbial targets [81]. These physical features directly affect catalytic activity, detection sensitivity, and the efficiency of signal transduction, making morphology control a key design parameter in MOF-based biosensors [82].

Precise size reduction is one strategy to enhance performance. Usman et al. demonstrated that downsizing MOF crystals to below 100 nm significantly improved their surface area, pore accessibility, and dispersibility under physiological conditions, which are factors crucial for biosensing in biological matrices [83]. Such nanoscale control can be achieved via bottom-up (e.g., modulation of reaction time, temperature, or solvent polarity) and top-down (e.g., mechanical grinding or sonication) approaches. These methods regulate MOF nucleation and growth kinetics, enabling uniform particle size, enhanced crystallinity, and biocompatibility for practical sensor deployment.

**Figure 4:**
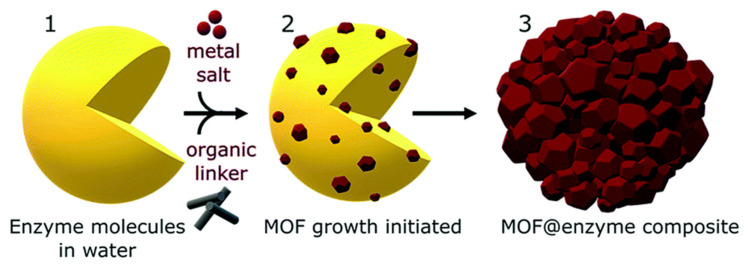
Illustration of the biomimetic mineralization process involved in the growth of ZIF-8 on enzyme surfaces. 1, 2, and 3 correspond to the raw enzyme, the growth of MOF crystals on the enzyme, and the final composite material in which the enzyme is fully encapsulated by the MOF, respectively. Copyright 2021, RSC [84].

Morphology engineering also plays a vital role in nanozyme-based microbial detection. Singh et al. utilized a biomimetic mineralization strategy to encapsulate enzymes such as glucose oxidase (GOx), organophosphorus hydrolase (OpdA), and α-chymotrypsin within ZIF-8 nanocrystals (Figure 4) [84]. The resulting ZIF-8@enzyme composites exhibited uniform morphology and high structural stability, preserving enzymatic activity under harsh conditions. These composites were integrated into electrochemical biosensors, where their nanoscale features facilitated efficient electron transfer and robust microbial detection with high sensitivity and selectivity.

Morphology optimization further enhances electrocatalytic properties in pathogen-related sensing. Nataraj et al. synthesized ZIF-67 using various methods such as co-precipitation, autoclaving, and hydrothermal synthesis to explore morphology-performance correlations in a dual-mode sensing platform [85]. Among the variants, ZIF-67-C exhibited the most favorable characteristics, including a uniform rhombic-dodecahedral morphology and minimal aggregation, which contributed to superior electrocatalytic activity and peroxidase-like behavior. This morphology enhanced the adsorption of 3,3′,5,5′-tetramethylbenzidine (TMB) and hydroxyl radical (•OH) generation from H_2_O_2_, enabling efficient substrate oxidation and high signal output. ZIF-67-C achieved low detection limits (0.014 μM electrochemical, 0.034 μM colorimetric) and demonstrated high recovery in real water samples, reinforcing the importance of morphology optimization for sensitive biosensing. This enhanced morphology improved the detection of model substrates and demonstrated the importance of synthesis-controlled morphology for biosensor performance in aqueous samples.

Additionally, Wang et al. reported a rapid synthesis of CdTe@ZIF-8 core–shell nanocomposites with tunable morphology and pore size for selective molecular detection [86]. The ZIF-8 shell functioned as a size-selective membrane, allowing the passage of small analytes (e.g., hydrogen peroxide) while excluding larger biomolecules. This design enabled fluorescence quenching only by reaction intermediates, facilitating sensitive detection of enzymatic activity from uricase and GOx. The ability to selectively control molecular transport based on MOF pore architecture highlights the utility of morphology design in microbial biosensing, where size exclusion is vital for specificity.

### 3.4. Composite and Hybrid Structures

The integration of MOFs with functional materials such as carbon nanotubes, graphene oxide, metal nanoparticles, and conductive polymers has emerged as a powerful strategy to overcome the inherent limitations of pristine MOFs, including low conductivity and limited stability [87]. These hybrids often demonstrate improved signal transduction, environmental durability, and broader functionality, making them ideal candidates for developing robust and multifunctional microbial biosensors [27,88].

A compelling example is the development of a copper-based hybrid nanozyme, ML-Cu_2_O@Cu-MOF, which incorporates Cu_2_O nanocrystals into a mixed-ligand MOF [89]. This structure hosts multiple copper valence states (Cu^0^/Cu^+^/Cu^2+^) and defect-rich domains, significantly improving electrochemical conductivity and aptamer immobilization. Integrated into an aptasensor for *Staphylococcus aureus* detection, the composite achieved ultralow detection limits (2.0 CFU m/L via EIS and 1.6 CFU m/L via DPV) with strong stability in food samples. This example highlights the potential of multivalent metal composites for ultrasensitive microbial pathogen monitoring.

Hybrid MOF systems have also demonstrated clinical relevance. A Zr-based MOF-enabled aptasensing platform was designed for the selective detection of *Acinetobacter baumannii* in blood [90]. The strategy employed Zr-MOFs for two complementary roles: magnetic enrichment via Zr-coated Fe_3_O_4_ nanoparticles (Zr-mMOF) and fluorescence amplification via fluorescein-loaded MOFs (F@UIO-66-NH_2_). Two aptamer-functionalized probes targeting bacterial surface markers and lipopolysaccharides formed a sandwich complex, and phosphate-induced MOF decomposition amplified the signal. This platform achieved detection as low as 10 CFU mL^−1^ within 2.5 h, with >90% aptamer loading and high recovery efficiency, demonstrating strong potential for bloodstream infection diagnostics.

To tackle the challenge of multidrug-resistant bacteria (MDRB), a hybrid nanozyme platform combining an MOF–COF (covalent organic framework) composite with boric acid functionality and a DNA-based multivalent aptamer scaffold was developed [91]. The hybrid system exhibited dual-mode peroxidase-like activity and high microbial affinity, enabling the fluorescence and colorimetric detection of *A. baumannii* and *Pseudomonas aeruginosa* down to 2–3 CFU m/L. The sensor maintained excellent performance in complex biological fluids, including serum, cerebrospinal fluid, and urine, emphasizing its clinical utility.

**Figure 5:**
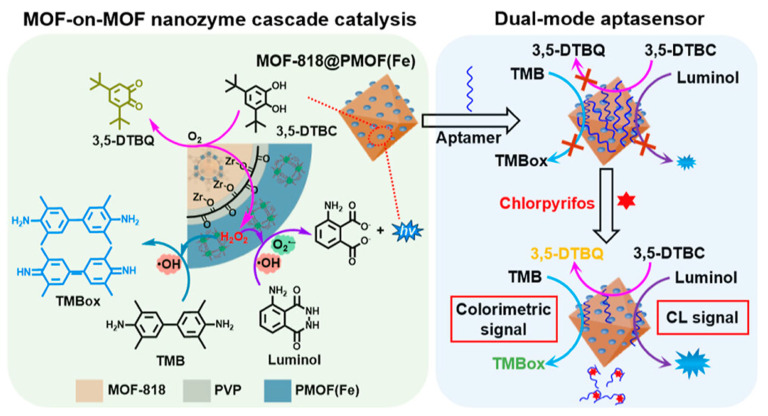
Depiction of the catalytic mechanism and aptasensing application employing an MOF-on-MOF nanozyme system. Copyright 2023, ACS [92].

Another sophisticated design featured an MOF-on-MOF nanozyme architecture (MOF-818@PMOF(Fe)) with cascade catalytic activity [92]. In this configuration, MOF-818 generated H_2_O_2_ in situ, which was subsequently utilized by PMOF(Fe) to produce ROS, enabling amplified chemiluminescence and colorimetric signals (Figure 5). When combined with a chlorpyrifos-specific aptamer, this dual-catalyst system achieved high sensitivity and selectivity in microbial sensing scenarios, demonstrating the power of rationally engineered hybrid nanozymes.

**Figure 6:**
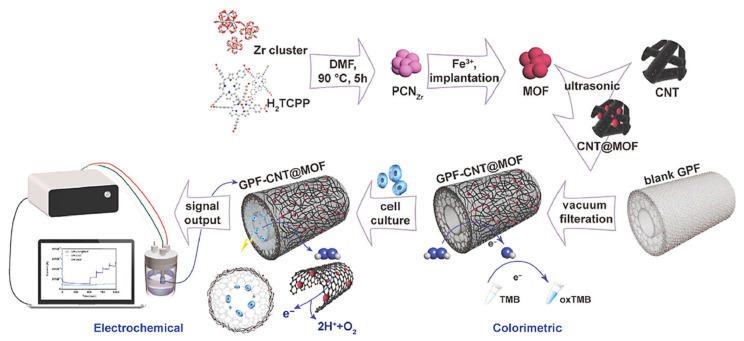
Diagram outlining the main stages in the preparation of GPF-CNT@MOF electrodes and the underlying sensing mechanism. Copyright 2024, Elsevier [93].

Beyond detection, composite systems have enabled the real-time monitoring of microbial metabolism. Ma et al. developed a 3D gradient porous fiber (GPF) membrane functionalized with CNT/MOF composites for the direct detection of H_2_O_2_ secreted by live cells (Figure 6) [93]. CNTs enhanced conductivity and MOF dispersion, while the MOF component served as a stable peroxidase mimic. This integrated scaffold allowed nonenzymatic, high-performance biosensing with excellent stability, offering a valuable platform for microbial metabolic profiling and diagnostics.

Minimally invasive diagnostics have also benefited from MOF hybrids. Zhao et al. introduced a microneedle-based biosensor using swellable hydrogel tips loaded with a DNAzyme@MOF composite for uric acid detection [94]. The DNAzyme enhanced the peroxidase-like activity of the MOF, enabling sensitive colorimetric analysis of trace analytes in interstitial fluid without relying on natural enzymes. This wearable hybrid platform exemplifies the growing biomedical relevance of MOF-based composites for microbial-related analyte monitoring.

Nanoparticle integration further improves MOF sensor performance. Guan et al. developed a composite nanozyme (Fe_3_O_4_@Au/MOF) by incorporating magnetic Fe_3_O_4_ and conductive Au nanoparticles into the MOF matrix [95]. The dual-nanoparticle system enhanced both recyclability and electron transfer, producing a highly sensitive electrochemical sensor for p-aminophenol with excellent selectivity and stability. Such platforms demonstrate how multifunctional nanocomposites can be tailored for efficient and reusable microbial biosensing.

In another example, a stimuli-responsive MOF/polymer hybrid was created by grafting dual-responsive poly(N-2-dimethylaminoethyl methacrylate) (PDM) onto UiO-66-NH_2_ scaffolds [96]. This “soft cage” encapsulated GOx and horseradish peroxidase (HRP), providing a dynamic microenvironment that responded to pH and temperature changes. The hybrid enhanced catalytic efficiency nearly ninefold and enabled colorimetric glucose detection in rat serum, underscoring the potential of MOF–polymer composites for adaptive biosensing in complex microbial contexts.

In summary, a variety of rational design strategies have been employed to enhance the enzyme-mimicking properties of MOF nanozymes for microbial biosensing. These strategies include active center engineering, pore environment modulation, surface functionalization, and hybridization with other functional materials. Each approach offers distinct advantages in improving catalytic efficiency, sensitivity, and target selectivity. Table 2 summarizes representative examples of these MOF nanozyme design strategies, highlighting the target microorganisms, type of enzyme-mimetic activity, detection methods, and associated benefits.

## 4. Detection Mechanisms and Signal Transduction

The effectiveness of MOF nanozymes in microbial biosensing relies not only on their design but also on the detection mechanisms employed [97]. Signal transduction converts microbial presence into quantifiable outputs, and MOFs can facilitate this through catalytic reactions that generate optical, electrochemical, or fluorescent signals [98,99].

### 4.1. Colorimetric Detection

Colorimetric sensing leverages the catalytic oxidation of chromogenic substrates such as TMB (3,3′,5,5′-tetramethylbenzidine) by MOF nanozymes in the presence of microbial analytes or their metabolic byproducts [100]. This method offers a simple, cost-effective, and visible output, suitable for point-of-care or field-based microbial detection.

Teymouri et al. embedded a Cu-MOF into an agar matrix to create Cu-MOF@AF, a multifunctional film that responded colorimetrically to microbial spoilage indicators such as hydrogen sulfide (H_2_S) and ammonia (NH_3_) [101]. This film not only exhibited enhanced mechanical properties (e.g., stretchability, water resistance, and UV blocking) but also showed potent antibacterial activity against *Escherichia coli* and *Staphylococcus aureus*. More importantly, the film served as a colorimetric sensor with a strong and irreversible response to volatile spoilage gases like hydrogen sulfide (H_2_S) and ammonia (NH_3_). Owing to the high surface area and active site availability of the Cu-MOF, the film achieved low limits of detection (LOD) and quantification (LOQ), while its dynamic and multi-hued color transitions allowed precise correlation with microbial spoilage indicators such as pH, total volatile basic nitrogen (TVB-N), and total viable count (TVC). Therefore, with this colorimetric platform, the real-time monitoring of food freshness and microbial contamination could be achieved.

Building on the need for selective detection of bacterial pathogens, Wang et al. designed a Cu-MOF nanoparticle (~550 nm) with intrinsic peroxidase-like activity that catalyzed the oxidation of TMB in the presence of hydrogen peroxide (H_2_O_2_), resulting in a visible yellow color change [102]. This construct catalyzed TMB oxidation in the presence of H_2_O_2_, resulting in a distinct yellow color change. The aptamer-functionalized MOF enabled the selective recognition and magnetic separation of *Staphylococcus aureus*, demonstrating its applicability for targeted pathogen detection.

For enhanced selectivity toward viable bacteria, Li et al. integrated a bacteriophage (SapYZUs8) onto a Cu-MOF nanozyme to construct a biosensor for detecting viable *Staphylococcus aureus* in food matrices [103]. The resulting SapYZUs8@Cu-MOF hybrid enabled visual detection within 30 min and demonstrated high specificity and a low detection limit of 1.09 × 10^2^ CFU/mL. The sensor remained effective even in complex matrices such as pork and milk, underscoring its applicability for real-world food safety monitoring.

In efforts to improve both accuracy and versatility, Wang et al. developed a dual-mode biosensor integrating both colorimetric and electrochemical outputs for the detection of Vibrio parahaemolyticus [104]. This sensor employed phenylboronic acid-modified CuO_2_ nanodots embedded within an MOF (CP@MOF), which facilitated hydroxyl radical generation under acidic conditions, oxidizing TMB and producing a visible color change. The system also allowed Cu^2+^ ion-based electrochemical quantification, achieving a combined detection accuracy of 88.7%, thus offering an effective strategy for on-site seafood pathogen monitoring.

**Figure 7:**
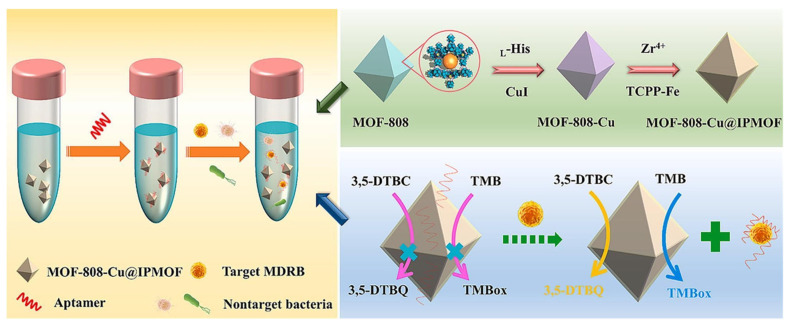
Standard procedure for colorimetric detection of MDRB and associated sensing techniques. Copyright 2025, Elsevier [105].

To combat challenges associated with multidrug-resistant (MDR) pathogens, Li et al. engineered a self-cascade MOF@MOF nanozyme consisting of Cu-functionalized MOF-808 and iron porphyrin MOFs [105]. This cascade system mimicked both catechol oxidase and peroxidase activities, enabling in situ H_2_O_2_ generation followed by amplified TMB oxidation (Figure 7). Coupled with specific aptamers, the system achieved the ultrasensitive detection of MRSA and *Pseudomonas aeruginosa* with detection limits as low as 5 and 2 CFU/mL, respectively. The synergistic catalytic effect of the hybrid nanozyme provided a highly specific and sensitive tool for MDR bacteria monitoring in both clinical and food settings.

To facilitate quick differentiation among bacterial species, Hao et al. fabricated a 2D Ni–Co bimetallic MOF (2D-NCM) that demonstrated enhanced peroxidase-like activity and allowed multichannel absorbance-based detection [106]. This material exhibited enhanced peroxidase-like activity and produced distinct, time-dependent absorbance changes upon interaction with different microbial species, including clinical pathogens and brewing fungi. The resulting multichannel detection profile enabled label-free, wash-free microbial discrimination within 30 min, making it suitable for high-throughput diagnostics in clinical and food environments.

**Figure 8:**
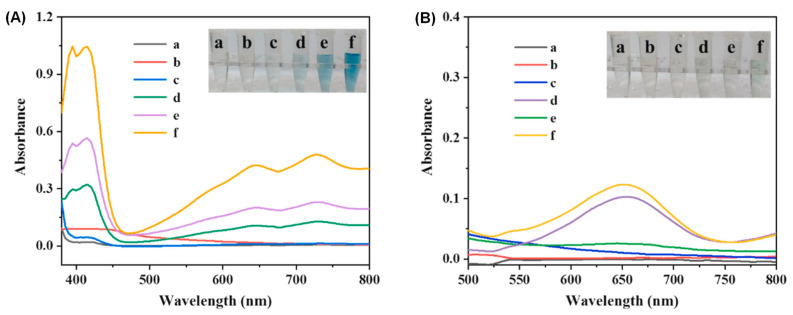
(**A**) UV–Vis absorption spectra comparing ABTS with various catalysts: (a) ABTS alone, (b) MVCM@β-CD, (c) ZIF-67 + ABTS, (d) 2D Co-MOF + ABTS, (e) MVCM + ABTS, and (f) MVCM@β-CD + ABTS. (**B**) UV–Vis absorption spectra of TMB under similar conditions: (a) TMB alone, (b) MVCM@β-CD, (c) ZIF-67 + TMB, (d) 2D Co-MOF + TMB, (e) MVCM + TMB, and (f) MVCM@β-CD + TMB, all measured in NaAc-HAc buffer (pH 3.0) after 10 min at room temperature (Inset: corresponding colorimetric changes captured in photographs). Copyright 2023, Elsevier [107].

Further advancing the versatility of MOF nanozymes in colorimetric sensing, Ren et al. synthesized a two-dimensional cobalt-based MOF (MVCM@β-CD) with enhanced oxidase-like activity, achieved by modulating the Co(III)/Co(II) ratio and surface functionalizing with β-cyclodextrin (β-CD) [107]. This rational design improved surface accessibility and catalytic efficiency, enabling the highly sensitive colorimetric detection of aminophenol isomers. Notably, while the system showed no background reaction with ABTS (Figure 8A), it generated a strong visual signal upon TMB oxidation (Figure 8B), achieving a detection limit of 0.16 μM for m-aminophenol. Although originally demonstrated with chemical contaminants, the catalytic and surface engineering strategies of MVCM@β-CD can be readily adapted to microbial sensing platforms, particularly for detecting pathogen-related metabolites or environmental pollutants linked to microbial activity. This highlights its potential for developing rapid, low-cost, and label-free biosensors relevant to microbial monitoring.

Collectively, these studies underscore the versatility and efficacy of nanozyme-based MOFs in colorimetric microbial detection. By combining catalytic activity with molecular recognition (via aptamers or phages) and innovative sensor platforms (films, cascades, or dual-mode systems), MOF-based nanozymes offer rapid, sensitive, and field-deployable tools for pathogen monitoring in food safety, clinical diagnostics, and environmental surveillance.

### 4.2. Electrochemical Detection

Electrochemical biosensors based on MOF nanozymes have gained traction in microbial diagnostics due to their sensitivity, portability, and ability to provide real-time readouts. These sensors often rely on redox reactions involving microbial byproducts (e.g., H_2_O_2_) or recognition events facilitated by aptamers or antibodies [108,109].

To address both the detection and treatment of MRSA, Mo et al. constructed a multifunctional nanozyme system using G-quadruplex/hemin DNAzyme-aptamer probes and tannic acid-chelated Au nanoparticle (Au-TA)-decorated Cu-based MOF nanosheets (GATC) [110]. This system integrates Cu-based MOF nanosheets with tannic acid-chelated gold nanoparticles (Au-TA) and G-quadruplex/hemin DNAzyme-aptamer probes. Among several MOF compositions, monometallic Cu-ZIFs showed superior catalytic and probe-loading efficiency over bimetallic alternatives such as CoCu-ZIF and ZnCu-ZIF. The hybrid system exhibited enhanced peroxidase (POD)-like activity for robust hydroxyl radical (•OH) production, along with catalase-like and GSH oxidase-like functionalities to alleviate hypoxia and minimize ROS scavenging, respectively. GATC enabled aptamer-mediated selective recognition of MRSA, achieving potent antibacterial activity at low concentrations (3 μg/mL). Furthermore, the platform supported both electrochemical and chromogenic detection modes, offering the real-time monitoring of infection treatment. This multifunctional construct exemplifies the integration of detection and therapeutic functionalities into a single MOF-based nanozyme system.

In another electrochemical strategy, Gao et al. reported an electrochemical platform specifically designed for the detection of H_2_O_2_ as a biomarker for microbial oxidative stress responses [111]. The system employed a synergistic hybrid of graphene (Gr) and MOF-on-MOF nanozymes composed of iron and copper (FeCu-NZs). Fe-MOFs were first synthesized via a solvothermal route and then decorated with Cu^2+^ ions to enhance their peroxidase-like catalytic efficiency. Integration with graphene further amplified the signal response due to improved conductivity. The FeCu-NZs-based sensor achieved a remarkable detection limit of 0.06 μM across a broad linear range (0.1–3800 μM) for H_2_O_2_. In addition to its electrochemical capabilities, this system enabled complementary colorimetric detection via hydroxyl radical generation and TMB oxidation. Application to real samples showed high recovery rates (>95.7%), underlining its utility for microbial monitoring in complex biological or environmental matrices. Given that H_2_O_2_ is a critical marker in host–pathogen interactions, especially under inflammatory or infection conditions, this system holds promise for diagnostic applications in microbiology and medicine.

**Figure 9:**
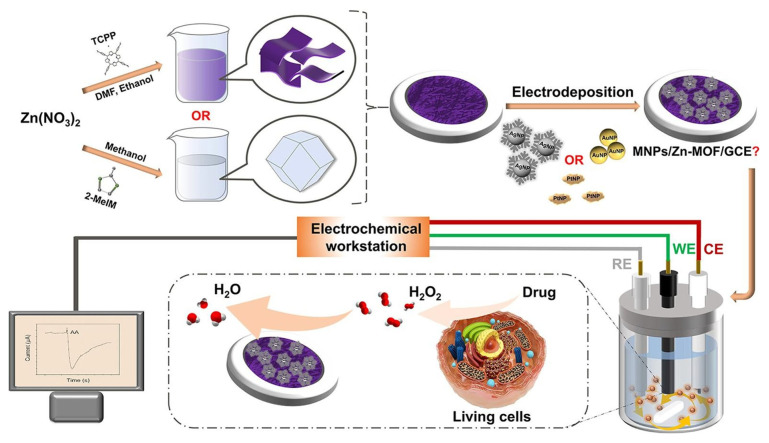
Illustration of the key steps involved in constructing MNPs/Zn-MOF-modified electrodes and their application in an H_2_O_2_ sensor for the real-time monitoring of H_2_O_2_ released by living cells upon drug stimulation, with data transmission to the electrochemical workstation. Copyright 2022, Elsevier [112].

To explore the role of MOF nanozymes in redox-active microbial sensing, Chen et al. developed a zinc-based MOF (Zn-MOF)-supported electrochemical platform, where metal nanoparticles were electrodeposited onto Zn-MOFs with varying morphologies to form a self-supported nanozyme system [112]. Among the tested nanoparticles, silver (AgNPs) exhibited superior electrocatalytic activity for hydrogen peroxide (H_2_O_2_) reduction compared to platinum (PtNPs) and gold (AuNPs). The study further revealed that two-dimensional Zn-MOFs (2D Zn-MOFs) offered higher surface area, better conductivity, and improved metal dispersion compared to their three-dimensional counterparts (3D Zn-MOFs), resulting in enhanced electrochemical performance. The Ag/2D Zn-MOF-modified electrode demonstrated a low detection limit of 1.67 μM and a wide linear range from 5.0 μM to 70 mM for H_2_O_2_ detection (Figure 9). This system was successfully employed to monitor H_2_O_2_ released by both normal and tumor cells during drug stimulation, demonstrating the potential of MOF-based nanozymes for real-time microbial and cellular redox monitoring.

Beyond electrochemical sensing, nanozyme MOFs also enable fluorescence and luminescence-based microbial detection, offering high sensitivity and visual readouts. The following section outlines key advances in this area.

### 4.3. Fluorescence and Luminescence

Fluorescence and luminescence-based microbial detection using MOF nanozymes offers dual-mode signal output and high analytical sensitivity [113]. These systems often exploit catalytic signal amplification in combination with optical reporters that respond selectively to microbial targets or associated molecules [114,115,116].

To target biofilm-associated infections, Liu et al. developed Ce-MOF nanozymes with dual DNase- and peroxidase-like activities [117]. The Ce(IV) complex degraded extracellular DNA (eDNA), a key structural biofilm component, while the MOF catalyzed H_2_O_2_ to eliminate released bacteria. This synergistic system effectively disrupted biofilms, inhibited bacterial recolonization, and promoted wound healing in vivo underscoring nanozyme-MOFs’ therapeutic potential.

For detecting microbial residues in food and clinical samples, Ye et al. engineered a ratiometric fluorescence sensor using a hemin-loaded MOF with intrinsic blue fluorescence and peroxidase activity [118]. Tetracycline enhanced peroxidase catalysis via π–π stacking with confined hemin, oxidizing TMB to a dark blue product. Concurrently, tetracycline aggregation triggered green fluorescence (λ_em_ = 535 nm) while quenching the MOF’s native blue emission (λ_em_ = 425 nm) via IFE. This enabled ratiometric fluorescence sensing (F_535_/F_425_), achieving detection limits of 27.2 nM (colorimetric) and 4.1 nM (fluorescent), showcasing excellent selectivity and on-site application potential. Therefore, nanozyme-based MOFs offer greater potential for structural modification, enabling the development of high-performance, multifunctional biosensors for microbial detection. A comprehensive comparison of the reported MOF nanozyme-based microbial biosensing strategies, including detection modes, nanozyme systems, target analytes, and performance metrics, is summarized in Table 3.

## 5. Biomedical Applications

In biomedical contexts, the rapid and accurate detection of pathogenic microbes is critical for early diagnosis, infection control, and treatment monitoring [119]. MOF nanozymes offer significant promise due to their tunable biorecognition capabilities and efficient signal generation [120].

### 5.1. Pathogen Detection in Clinical Samples

The rapid and accurate detection of clinically relevant bacteria including *Escherichia coli*, *Staphylococcus aureus*, and multidrug-resistant strains such as MRSA remains a significant challenge in clinical diagnostics [121]. MOF-based nanozymes have emerged as promising platforms due to their tunable surface chemistry, high surface area, and catalytic capabilities. These features allow them to be engineered for the selective recognition of microbial biomarkers or toxins, enabling rapid and sensitive detection in various clinical matrices such as blood, urine, saliva, and wound exudates [122]. Moreover, their compatibility with existing clinical workflows facilitates early disease diagnosis and timely therapeutic intervention [123].

The global rise in antimicrobial resistance, particularly among pathogens like MRSA, underscores the urgent need for advanced diagnostic and therapeutic technologies. Traditional antibiotics such as vancomycin are increasingly undermined by bacterial adaptations, including metabolic tolerance mechanisms driven by the accumulation of toxic byproducts. To address these issues, Wang et al. developed a multifunctional gallium/copper-based MOF (Ga/Cu-MOF) nanozyme designed for both pathogen detection and eradication [124]. This nanozyme leverages the structural advantages of MOFs to co-deliver antibiotics and release Ga^3+^ and Cu^2+^ ions in a controlled manner. These ions disrupt bacterial metabolism by suppressing nitric oxide synthesis and promoting the degradation of S-nitrosothiols, thereby weakening the bacteria’s stress response systems. It not only enhances antibiotic susceptibility but also induces programmed bacterial cell death via ferroptosis and cuproptosis. The Ga/Cu-MOF nanozyme also demonstrated excellent antibacterial activity by disrupting biofilms and eliminating intracellular bacteria residing within host immune cells. When tested in clinically relevant infection models, including skin wounds and osteomyelitis, the nanozyme effectively reduced bacterial burden, mitigated inflammation, and promoted tissue healing, highlighting its potential for integrated diagnostic and therapeutic applications in infectious disease management.

**Figure 10:**
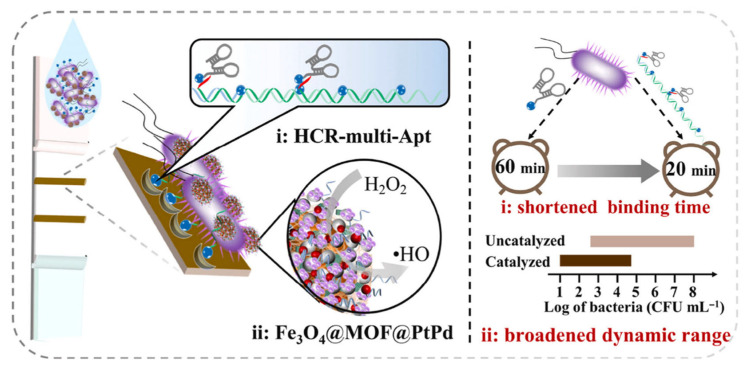
Illustration of the HCR-multi-Apt assembly process and its application in bacterial detection. Copyright 2024, Elsevier [125].

To overcome limitations in traditional lateral flow assays (LFAs) such as low sensitivity and the need for amplification steps, Li et al. designed a next-generation detection platform by combining a rationally engineered multivalent aptamer (multi-Apt) targeting *Staphylococcus aureus* with a multifunctional nanozyme, Fe_3_O_4_@MOF@PtPd, functionalized with vancomycin [125]. The optimized aptamer significantly improved binding affinity (from 135.9 nM to 16.77 nM), and the resulting MA-MN LFA system enabled magnetic capture, signal amplification, and simplified the detection of *Staphylococcus aureus* (Figure 10). This ternary complex-based assay achieved ultra-sensitive detection, with a limit of just 2 CFU/mL and a dynamic range spanning from 10 to 1 × 10^8^ CFU/mL all within 30 min. Its simplicity, speed, and high specificity make it well-suited for real-time clinical diagnostics, particularly for Gram-positive bacterial infections. Together, these advances underscore the versatility and promise of MOF-based nanozyme platforms in the clinical setting not only for rapid and accurate pathogen detection but also for integrated antimicrobial strategies that address both diagnosis and treatment in the era of rising antibiotic resistance.

### 5.2. Real-Time and POC Diagnostics

The development of portable, user-friendly biosensors is vital for decentralized healthcare [126]. MOF nanozymes enable real-time monitoring through catalytic reactions that produce immediate colorimetric or electrochemical signals [127]. These sensors are especially valuable in resource-limited settings or during outbreaks where rapid screening is essential.

Bacterial infections remain a major public health concern, necessitating rapid and highly sensitive diagnostic tools for timely intervention. To address this, Ren et al. reported that a smart, dual-mode biosensor was developed using nanozyme-based MOFs for the point-of-care detection of pathogenic bacteria [128]. This innovative sensor integrates a SERS platform with oxidase-like nanozyme activity, enabling both colorimetric and spectroscopic detection. The system comprises two key components: an iron oxide nanoparticle-based capture module functionalized with specific aptamers for bacterial recognition and enrichment and a signal module composed of mesoporous MnO_2_ nanozymes loaded with plasmonic gold nanoparticles. Functionalized with concanavalin A, the signal module creates strong SERS hotspots and catalyzes the oxidation of non-SERS-active TMB into a highly active oxTMB form, producing both a strong Raman signal and a visible color change. This dual readout enhances detection reliability, with limits as low as 7 CFU/mL for SERS and 30 CFU/mL for colorimetric analysis. The sensor demonstrates excellent specificity for *Staphylococcus aureus* and performs effectively in complex clinical samples such as sepsis-infected blood. These features underscore the promise of nanozyme MOF-based smart sensors for real-time, on-site microbial diagnostics in clinical applications.

**Figure 11:**
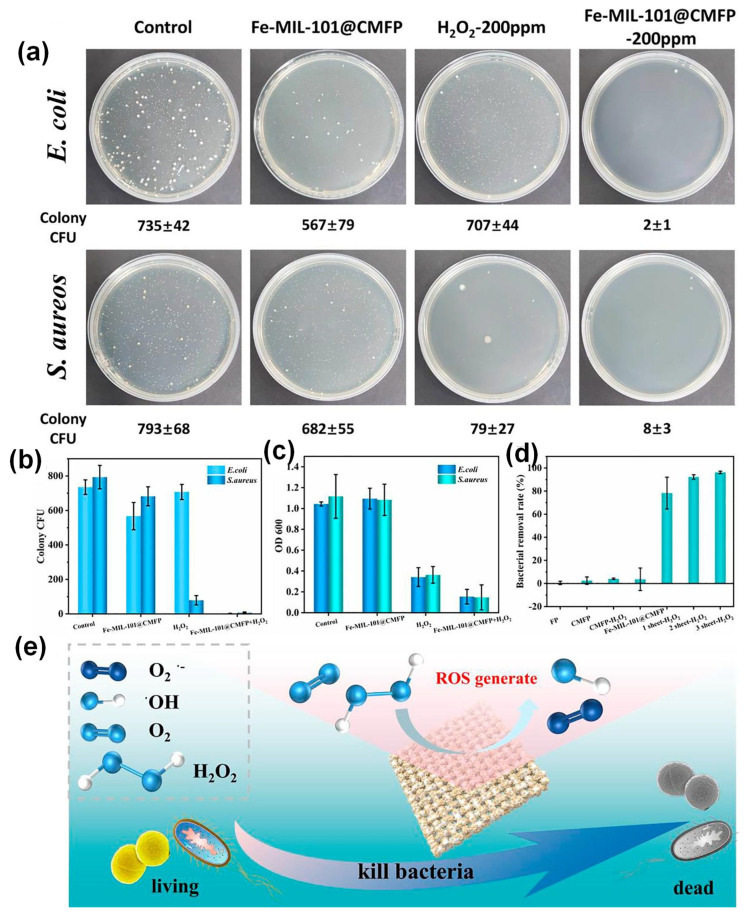
(**a**) Bacterial culture results on agar plates following treatment with Fe-MIL-101@CMFP. (**b**) Quantification of bacterial colonies. (**c**) OD600 measurements of the filtrate over a 12 h period. (**d**) Comparison of sterilization efficiency across different filter paper materials. (**e**) Schematic illustration of the bacterial killing mechanism. Copyright 2024, Elsevier [129].

Access to clean water is vital for human health, yet underdeveloped regions and emergency scenarios often lack efficient, low-energy water purification methods. To address this challenge, Fu et al. reported that a novel class of portable disinfection materials was developed by integrating Fe-MIL-101 nanozyme metal–organic frameworks (MOFs) onto flexible carboxymethyl cellulose substrates (e.g., filter paper, cotton, gauze) through in situ cross-linking growth [129]. These Fe-MIL-101@fiber composites function as point-of-use (POU) water disinfection modules, offering strong antibacterial activity, ease of transport, and biosafety (Figure 11). Among them, the Fe-MIL-101@carboxymethyl filter paper (Fe-MIL-101@CMFP) demonstrated exceptional performance, maintaining structural integrity and filtration efficiency even after 200 cycles of manual or mechanical stress. The composite exhibits robust oxidase- and peroxidase-mimicking activity, producing ROS such as hydroxyl radicals (•OH) and superoxide (O_2_•^−^), which enable rapid and efficient bacterial elimination (~99%). Notably, the material is effective against microbes present in real field water without inducing biosafety concerns. These flexible, nanozyme-powered MOF composites show great promise for decentralized microbial disinfection, offering a practical and safe solution for water sterilization in resource-limited or emergency healthcare settings.

Sensitive, portable, and non-invasive methods to detect pathogenic bacteria are crucial for POC diagnostics. Feng et al. developed an advanced ECL biosensor by combining a flexible paper-based sensor with a disposable three-electrode detection system. Cellulose paper was functionalized to specifically respond to *Staphylococcus aureus* by using an aptamer as the recognition element and a probe DNA linked to GOx (probe DNA-GOD) for signal amplification [130]. When the aptamer binds to *Staphylococcus aureus*, it triggers the controlled release of probe DNA-GOD. The residual probe DNA-GOD on the paper sensor reacts with glucose, causing a notable decrease in the ECL signal. To enhance the biosensor’s ECL performance, numerous Ru(bpy)_3_^2+^ molecules were incorporated into porous zinc-based metal–organic frameworks (MOFs) to create Ru(bpy)_3_^2+^-functionalized MOF nanoflowers (Ru-MOF-5 NFs). This biosensor allows for the precise, non-destructive, and real-time detection of *Staphylococcus aureus* contamination in food samples, offering a novel approach for sensitive bacterial identification (Table 4).

### 5.3. Wound Monitoring and Infection Control

Chronic wounds, particularly in diabetic patients, are highly susceptible to microbial colonization and persistent infections, which significantly impede the healing process and increase the risk of complications [140]. These infections are often compounded by the presence of bacterial biofilms and rising antibiotic resistance, necessitating the development of multifunctional wound care systems capable of both real-time infection monitoring and active therapeutic intervention [131]. When integrated into wound dressings or hydrogels, MOF nanozymes can provide a dual function, sensing infection-associated biomarkers such as hydrogen peroxide (H_2_O_2_) or glutathione and simultaneously catalyzing therapeutic reactions that neutralize pathogens and oxidative stress [141].

A notable example of such a multifunctional platform is the nanozyme reported by Zhao et al., in which gold nanoclusters (Au NCs) were anchored onto zirconium-based porphyrin MOFs (Au NCs@PCN) via in situ growth [142]. The resulting hybrid nanozyme exhibited both strong reactive oxygen species (ROS) generation and photothermal effects under near-infrared (NIR) irradiation, achieving localized heating up to 56.2 °C. This synergistic response enabled efficient bacterial membrane disruption and protein leakage, with bactericidal rates of 95.3% against MRSA and 90.6% against ampicillin-resistant *Escherichia coli*. Moreover, in diabetic wound models, Au NCs@PCN treatment significantly accelerated healing, reducing wound size to just 2.7% within 21 days. Immunoblot analysis further demonstrated the increased expression of angiogenic and epithelial proliferation markers, highlighting the platform’s regenerative potential.

**Figure 12:**
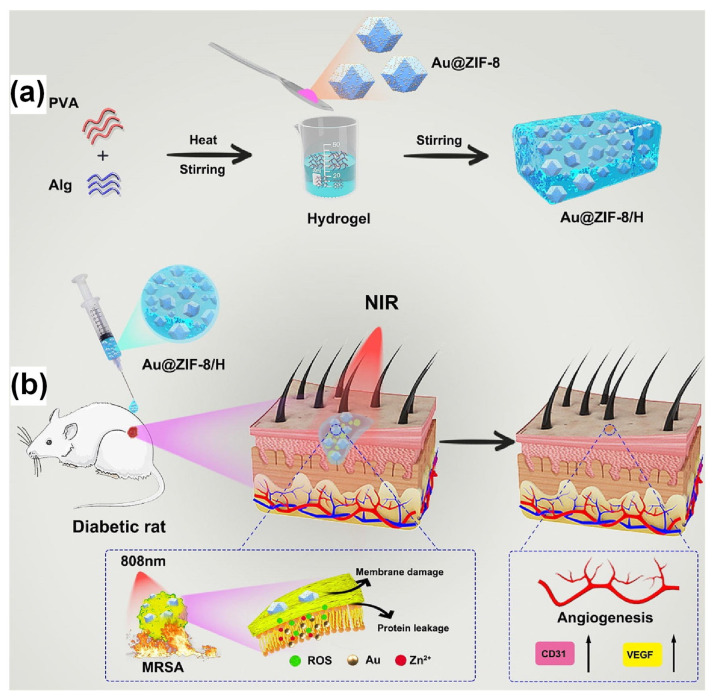
(**a**) Illustration of the synthesis procedure for the Au@ZIF-8/H composite. (**b**) Diagram showing its antibacterial action, stimulation of cellular activity, and promotion of skin cell and epidermal regeneration to enhance the healing of diabetic infected wounds. Copyright 2023, Elsevier [143].

Building on this concept, Ren et al. developed a peroxidase-mimicking nanozyme by integrating Au NCs into a zeolitic imidazolate framework (Au@ZIF-8) [143]. This nanozyme exhibited enhanced peroxidase-like activity and photothermal effects upon NIR exposure, leading to effective bacterial membrane disruption (66.3%) and MRSA clearance (up to 97%). The platform catalyzed the conversion of endogenous H_2_O_2_ into hydroxyl radicals (•OH), facilitating both bacterial eradication and biofilm degradation (Figure 12). The in vivo application of Au@ZIF-8 resulted in a remarkable wound closure rate of 97.4%, underscoring its suitability for chronic wound care where biofilm-associated infections are prevalent.

To address another critical aspect of wound-healing oxidative stress, Chao et al. proposed a smart antioxidative hydrogel system (MOF/Gel) comprising an MOF nanozyme embedded in a biocompatible gel matrix [132]. The nanozyme mimicked the activity of natural antioxidant enzymes, continuously scavenging excess ROS and restoring redox homeostasis. This biochemical regulation facilitated the transition of the wound environment from an inflammatory to a proliferative phase, leading to enhanced tissue regeneration. In comparative studies, a single application of MOF/Gel demonstrated healing efficacy comparable to that of clinically used epidermal growth factor (EGF) gels, further validating its therapeutic potential.

To combat the increasing threat of antibiotic resistance, Liu et al. engineered an antibiotic-free nanozyme system by growing ultrasmall AuNPs in situ on MOF-stabilized Fe_3_O_4_ NPs (Fe_3_O_4_@MOF@Au, or FMA NPs) [144]. These hybrid nanozymes exhibited robust POD-like activity even at low H_2_O_2_ concentrations (0.97 mM), producing bactericidal •OH radicals. The FMA-NPs showed strong antibacterial efficacy against both Gram-positive and -negative bacteria, with excellent biocompatibility. In vivo studies confirmed their wound-healing acceleration capabilities, demonstrating that MOF-based nanozymes can effectively circumvent the limitations of conventional antibiotics.

In addition to therapeutic efficacy, real-time infection monitoring is crucial for timely clinical intervention. Le et al. addressed this need by developing a dual-functional hydrogel composed of lysozyme-based hemin-loaded nanoparticles (Ly@He NPs) co-encapsulated with silver nitrate (AgNO_3_) in a double-network hydrogel matrix of gelatin and polyvinyl alcohol [133]. This system combined sustained antibacterial action mediated by silver ion release and nanozyme-catalyzed ROS production with colorimetric biosensing capabilities. Specifically, the peroxidase-like activity of Ly@He NPs enabled the hydrogel to detect glutathione, a bacterial infection biomarker, via visible color change. Both in vitro and in vivo results validated the hydrogel’s dual functionality in infection control and dynamic infection monitoring, paving the way for the next generation of smart wound dressings that integrate diagnostics with on-demand therapy. Therefore, by integrating catalytic activity with infection-responsive sensing and therapeutic features, these advanced materials offer a multifaceted solution to the pressing challenges of wound healing, especially in the context of diabetic ulcers and drug-resistant infections.

## 6. Environmental Applications

The environmental monitoring of microbial contamination is vital for water safety, air quality, and soil health. MOF nanozyme-based sensors offer portable, selective, and rapid detection solutions that can be deployed in diverse ecological settings. This section explores how these systems are adapted for environmental biosensing.

### 6.1. Waterborne Pathogen Detection

Waterborne pathogens such as *Escherichia coli*, *Salmonella*, and *Legionella* are major contributors to drinking water contamination, posing severe public health risks and environmental concerns [145]. The early and reliable detection of these pathogens is vital to prevent outbreaks and ensure safe water supplies. Traditional culture-based microbiological methods are often inadequate due to their slow turnaround time and inability to detect viable but non-culturable (VBNC) organisms. While molecular techniques such as RT-PCR and ELISA offer improved sensitivity, they are generally labor-intensive, costly, and not well-suited for on-site or field-based applications [146].

To overcome these limitations, nanozyme-based metal–organic frameworks (MOFs) have emerged as promising tools for waterborne pathogen detection. These hybrid materials combine the high surface area, porosity, and chemical tunability of MOFs with the catalytic (enzyme-like) activity of nanozymes, thereby facilitating efficient signal amplification in biosensing platforms [147]. MOF-based nanozymes have been effectively integrated into optical and electrochemical sensors, enabling rapid, sensitive, and cost-effective detection of pathogens such as *Escherichia coli* O157:H7 in contaminated water (Table 4). Their compatibility with portable systems, including smartphone-based readouts, further enhances their applicability for point-of-care and real-time environmental monitoring.

An illustrative example of this potential is the work by Magar et al., who developed a portable biosensing platform for the rapid and selective detection of *Staphylococcus aureus*, a common waterborne and foodborne pathogen [134]. This system employed copper-based MOFs (Cu-MOFs) integrated with gold nanoparticles (AuNPs) and mercaptopropionic acid (MPA) to immobilize specific antibodies onto a screen-printed electrode (SPE). The resulting disposable sensor (anti- *Staphylococcus aureus*@MPA/AuNPs-Cu-MOF@SPE) demonstrated a broad detection range from 10 to 10^7^ CFU/mL and a low detection limit of 0.132 CFU/mL, with high selectivity against non-target bacteria. Impressively, the entire sensing process was completed within 20 min, showcasing its effectiveness for on-site pathogen detection in water samples.

In another notable study, Zhao et al. synthesized a europium-based MOF, [Eu(DHBDC)_1.5_(DMF)_2_]·DMF, which exhibited high thermal and water stability along with ligand-based fluorescence at 506 nm [148]. Although the material lacked initial Eu^3+^-centered emission due to an energy mismatch with the ligand (2,5-dihydroxyterephthalic acid, DHBDC^2−^), the introduction of 2,6-pyridinedicarboxylic acid (DPA), a biomarker of Bacillus anthracis, restored the red fluorescence of Eu^3+^ at 593, 618, and 699 nm. This interaction produced a dual-emission ratiometric signal with a detection limit as low as 1.3 μM and strong anti-interference capabilities.

Moreover, recent insights into microbial signaling pathways further emphasize the importance of advanced detection tools. *Legionella pneumophila*, the causative agent of Legionnaires’ disease, utilizes a quorum-sensing molecule known as *Legionella* autoinducer-1 (LAI-1) for interspecies and interkingdom communication. Fan et al. demonstrated that LAI-1, synthesized by the enzyme *lqsA*, is secreted through outer membrane vesicles (OMVs), which facilitate both microbial signaling and host–pathogen interactions [135]. Using a Vibrio cholerae luminescence reporter and LC-MS/MS, LAI-1 was detected in OMVs derived from both *Escherichia coli* and *Legionella pneumophila* expressing *lqsA*. These OMVs activated bacterial signaling pathways and inhibited amoeba migration, indicating their role in environmental virulence. Furthermore, the overexpression of *lqsA* enhanced LAI-1 secretion, OMV production, and intracellular replication of *Legionella pneumophila* in macrophages. These findings underscore OMVs as vehicles for pathogen-derived biomolecules and advocate for the development of MOF-based nanozyme sensors targeting quorum-sensing signals for the effective monitoring of waterborne microbial threats.

### 6.2. Soil and Air Microbial Sensing

MOF-based nanozymes have emerged as powerful tools for monitoring and modulating microbial activity in soil and air environments, owing to their unique features such as high surface area, tunable porosity, and intrinsic enzyme-mimicking properties. These materials play a critical role in environmental remediation, microbial detection, and pollutant degradation.

Su et al. demonstrated the application of Mn_3_O_4_ nanozyme-coated microbial consortia to remediate heavy metal–polluted paddy soils. By enhancing the microbial reduction in toxic Cr^6+^ and promoting the immobilization of As^3+^, the nanozyme significantly reduced metal bioavailability, thereby lowering uptake by rice plants [149]. In the context of soil pesticide monitoring, Kumar et al. developed a nanozyme sensor array composed of carbon nanotubes decorated with various transition metals. This system could detect multiple pesticides in both soil and water samples with high sensitivity, showing detection limits in the range of 10.8–28.8 nM. The sensor array provided distinct colorimetric signatures for different analytes, allowing for easy field-level discrimination [150]. To mitigate cadmium (Cd) contamination in agricultural soils, Kong et al. introduced a microbial organic fertilizer (MOF) strategy. This approach boosted the population of iron-oxidizing bacteria in the rhizosphere, which facilitated iron plaque formation on rice roots. These plaques acted as a barrier to Cd uptake, effectively reducing Cd bioavailability in soil and its accumulation in rice grains [136].

For antibiotic residue detection, Ju et al. fabricated a Zn-based MOF sensor capable of selectively recognizing sulfasalazine, a common sulfonamide antibiotic. The sensor displayed a low detection limit of 31 nM and could be incorporated into paper-based test strips for on-site monitoring [151]. Additionally, Li et al. designed a cadmium-based MOF (Cd-MOF) capable of the dual fluorescent detection of both antibiotics and bacterial contaminants. This sensor achieved a limit of detection as low as 47 CFU/mL for Streptomyces albus, demonstrating its potential in environmental biosafety applications [152]. Addressing soil pollutants more broadly, Guselnikova et al. developed an MOF-5-based coating integrated with a plasmonic grating structure to serve as a highly sensitive SERS platform. This device allowed for the trace-level detection of organophosphorus compounds, achieving detection limits down to 10^−12^ M, highlighting its utility in chemical hazard surveillance [137].

For airborne microbial threats, Zhu et al. engineered nanofibrous membranes by integrating UiO-66-NH_2_ MOFs with quaternary ammonium groups. These membranes not only provided excellent filtration efficiency (>95%) against particulate matter but also exhibited potent antibacterial activity, making them suitable for high-performance air filters in healthcare and industrial settings [153]. Furthermore, Pan et al. reported a novel gas-phase biosensing platform based on cobalt-doped ZnO superparticles derived from MOFs. This material demonstrated high selectivity for detecting Listeria monocytogenes through its volatile biomarker, 3-hydroxy-2-butanone (3H-2B), offering a non-invasive and rapid detection method for foodborne pathogens in the air [154].

## 7. Challenges and Future Perspectives

MOF-based nanozyme microbial biosensors hold tremendous potential for highly sensitive and selective microbial detection. However, several critical challenges must be addressed before these systems can be widely adopted in practical applications [138].

First and foremost, the long-term stability and reusability of MOF nanozymes pose significant concerns. Many MOFs are prone to structural degradation and loss of catalytic activity after repeated usage or when exposed to harsh environmental conditions such as moisture, extreme pH levels, and microbial metabolites [155]. While innovative designs—like electrochemiluminescence biosensors incorporating covalently attached Ru complexes on NH_2_-MIL-53(Al)—have demonstrated enhanced durability and consistent performance, these remain exceptional cases rather than the norm [139]. To improve the reliability of MOF nanozymes, future research should prioritize enhancing their structural robustness and catalytic longevity. Strategies such as ligand engineering, post-synthetic modifications, and forming composites with highly stable materials like graphene or polymers are promising approaches to achieve this goal.

In addition to stability challenges, achieving high selectivity and specificity for target microbial strains remains an unmet need. Most current MOF nanozyme biosensors rely largely on generalized catalytic activity, which limits their ability to precisely identify microbial strains within complex biological matrices [156]. To overcome this limitation, integrating MOFs with selective biorecognition elements such as aptamers, antibodies, or molecularly imprinted polymers could significantly improve target specificity. Thanks to their tunable structures, large surface areas, and ease of surface modification, MOFs are ideally suited to develop sensors capable of highly selective microbial detection through mechanisms like photoinduced electron transfer (PET), resonance energy transfer (RET), and structural transformations [157].

Moreover, scaling up MOF production while maintaining quality and reducing costs remains a significant barrier to commercial viability. Although MOFs can be synthesized relatively straightforwardly in the laboratory, the transition to large-scale manufacturing is hindered by the need for high-purity metal precursors, energy-intensive synthesis, and complex purification processes. These factors not only increase production costs but also lead to batch-to-batch inconsistencies [158]. Therefore, future efforts should focus on developing green, low-cost, and scalable synthesis methods that ensure reproducibility and sustainability, making MOF nanozyme biosensors more economically feasible for widespread use.

Looking forward, the integration of MOF nanozymes into smart sensor platforms empowered by artificial intelligence (AI) and the Internet of Things (IoT) offers exciting prospects. Such integrated systems could facilitate the real-time, remote monitoring of microbial contamination in diverse settings including food safety, water quality, and healthcare environments. However, creating seamless interfaces between MOF materials and electronic components, data processing units, and wireless communication modules presents considerable technical challenges that require interdisciplinary innovation [159]. The tunable catalytic properties of MOFs combined with smart responsive materials provide a strong foundation for developing next-generation adaptive biosensors with enhanced sensitivity and selectivity.

Finally, ensuring the biosafety of MOF nanozyme biosensors and navigating the regulatory requirements are essential for real-world implementation. The use of novel nanomaterials in the clinical and environmental contexts requires comprehensive toxicity evaluations, biocompatibility assessments, and environmental impact studies [160]. Potential cytotoxicity and long-term safety must be rigorously examined [161]. Establishing standardized testing protocols and clear regulatory guidelines will be critical to guarantee safe application and foster public trust [162]. Without addressing these concerns, the translation of MOF-based biosensors from laboratory prototypes to practical tools will remain limited.

Therefore, tackling challenges related to stability, specificity, scalability, smart integration, and safety is vital for unlocking the full potential of MOF-based nanozyme microbial biosensors (Table 5). Through continued research and interdisciplinary innovation, these advanced biosensing platforms hold the key to revolutionize microbial detection, delivering sensitive, selective, cost-effective, and intelligent diagnostic solutions across a range of real-world applications.

## 8. Conclusions

Nanozymatic MOF-based biosensors have emerged as transformative platforms for highly sensitive and selective microbial detection, leveraging the intrinsic tunability of MOFs through structural alteration, composite formation, and the precise control of pore size, surface functionality, and metal centers to finely tailor their enzyme-mimicking catalytic activity, stability, and specificity. These engineered nanozymes offer rapid, cost-effective, and robust biosensing solutions that outperform conventional enzyme-based sensors in stability, reusability, and responsiveness, rendering them ideal for diverse biomedical and environmental applications. Integration with advanced signal transduction systems and smart diagnostic devices further enhances their capacity for the real-time, selective detection of pathogens and microbial metabolites. These multifunctional properties of MOF nanozymes as next-generation intelligent biosensing materials are critical for addressing challenges such as early disease diagnostics, antimicrobial resistance monitoring, and environmental pathogen surveillance. Efforts to improve biocompatibility, operational stability in complex biological and environmental matrices, and scalable synthesis methods are essential for accelerating clinical translation and widespread deployment, bridging the gap between laboratory innovation and impactful real-world applications. As immediate next steps, researchers should prioritize long-term stability testing under application-relevant conditions, the development of integrated, AI-compatible sensor prototypes, and the establishment of standardized protocols for biosafety evaluation and regulatory compliance.

## Figures and Tables

**Table 1:** Summary of key design and functional concepts in MOF-based nanozyme biosensing.

Key Concept	Description	Relevance to Biosensing	Representative Examples	Refs.
Structural Features of MOFs	MOFs are crystalline porous materials formed by metal ions/clusters and organic ligands, offering ultrahigh surface area, ordered networks, and tunable pore sizes.	Enable selective adsorption, diffusion of analytes, and high loading of active sites, forming the structural basis for biosensing and nanozyme integration.	Use of porous MOFs to concentrate microbial analytes or catalytic substrates.	[40,41,42,43,44]
Chemical Tunability of MOFs	MOFs can be customized at both the metal center and linker level, allowing the modulation of surface chemistry and catalytic activity.	Facilitates precise design of nanozyme activity, target specificity, and sensing selectivity.	Functionalized MOFs with redox-active metals or tailored linkers enhancing specificity for microbial detection.	[41,42,43,44]
Classification of Nanozymes	Nanozymes are classified into metal-based, carbon-based, and composite types based on material and catalytic mechanism.	Understanding classification aids in selecting the most suitable catalytic system for microbial biosensing applications.	FePPOP-1 for peroxidase-like sensing; Smutok et al.’s composite biosensor for L-lactate; cerium oxide nanozymes for ROS-related diagnostics; Mn_3_O_4_@g-C_3_N_4_ for pollutant sensing.	[45,46,47,48,49,50,51,52,53,54]
Advantages of MOFs as Nanozyme Hosts	MOFs offer high porosity, active site confinement, and chemical compatibility for hosting nanozymes.	Improves catalytic efficiency, stability, and substrate accessibility, enhancing detection sensitivity and robustness.	Use of MOFs to stabilize catalytic centers and provide porous diffusion pathways for microbial analytes.	[55,56,57]
Integration of MOFs and Nanozymes	Rational design of MOF–nanozyme hybrids leverages both structural and catalytic advantages, enabling multifunctional biosensing platforms.	Enhances sensitivity, selectivity, and potential for intelligent (e.g., point-of-care or responsive) microbial biosensing platforms.	Cerium oxide nanozymes within polymer-modified MOFs for ROS scavenging; multifunctional Mn_3_O_4_@g-C_3_N_4_ composites for pollutant sensing with enzyme-mimetic properties.	[53,54,58]

**Table 2:** Summary of MOF nanozyme design strategies for microbial biosensing.

Design Strategy	Target Microorganism	Type of Enzyme-mimetic Activity	Advantages	Representative Examples	Detection Method	Ref.
Active Center Engineering	Methicillin-resistant *Staphylococcus aureus* (MRSA)	Peroxidase-like	High catalytic activity; ultrasensitive detection; antibiotic susceptibility testing	Co_2_–O_10_ dual atomic sites in MOF-808; amplified chemiluminescence (~5800-fold)	Chemiluminescence	[64]
	*Salmonella* Typhimurium	Peroxidase-mimetic electrocatalysis	High sensitivity; synergistic catalysis via Cu, Zr, and AuNPs	AuNP-doped CuZr-MOF functionalized with DNA probes	Electrochemical	[65]
	Lipopolysaccharide (LPS) (endotoxin)	Dopamine oxidation catalysis	Ultra-low LOD; electrostatic recognition; high specificity	Cu^2+^-modified nanoscale MOFs	Electrochemical	[66]
	*Escherichia coli*, *Staphylococcus aureus*	Fluorescence modulation via surface interaction	Rapid detection; multiplex pathogen detection; applicable in food samples	Turn-on Fe-MOF fluorescence biosensor	Fluorescence	[67]
	*Staphylococcus aureus*	Peroxidase-like	Synergistic metal doping; enhanced catalytic activity	Amine-functionalized bimetallic Fe–Ni MOF-74	Colorimetric/Electrochemical	[68]
	—	Oxidase-like (ascorbate, glutathione, laccase)	Defect engineering enhances multi-enzyme mimicry; improved oxygen adsorption	Cysteine-deficient Co-based MOF (ZIF-L-Co)	Electrochemical (uric acid sensing)	[60]
	—	Peroxidase-like	Local pH regulation improves activity at physiological pH	PAA embedded in PCN-222-Fe	Colorimetric	[69]
Pore Environment Modulation	—	Peroxidase-like	Microenvironment tuning for physiological stability	PAA-modulated PCN-222-Fe	Colorimetric	[69]
Surface Functionalization	Multiple microbial species	Fluorescence quenching/recovery	Rapid microbial fingerprinting; high classification accuracy in complex matrices	2D-MOFs with fluorescent dye-labeled peptides	Fluorescence	[74]
	Bacteria (general)	ROS generation (photocatalytic antibacterial)	Dual detection and disinfection capability	Boronic acid-modified UiO-66 (Zr-UiO-66-B(OH)_2_)	Fluorescence	[75]
	*Escherichia coli*	Peroxidase-like	Rapid, specific detection; enhanced phage stability and signal amplification	NH_2_-MIL-101(Fe) conjugated with lytic bacteriophages	Colorimetric/Fluorescence	[76]
	*Pseudomonas aeruginosa*, *Escherichia coli*	Luminescent detection	Label-free, specific carbohydrate binding; stable in environment	Glycosylated NH_2_-MIL-53(Fe) with galactose/mannose ligands	Luminescence	[77]
	—	Dual-gated enzymatic activity	Programmable, tunable biosensing behavior	ZIF-8 functionalized with DNA surfactant micelles	Fluorescence/Enzymatic	[78]
	*Escherichia coli* O157:H7	Peroxidase-like	Wide detection range; improved electron transfer via polyaniline	Amino-functionalized MOF aptasensor	Electrochemical	[79]
	—	Fenton-like catalytic activity	Enhanced ECL signal; improved electron transfer	CoNi-MOF@PCN-224/Fe dual MOF-on-MOF system	Electrochemiluminescence	[80]
Hybridization with Other Materials	*Staphylococcus aureus*	Peroxidase-like	Improved conductivity and aptamer immobilization; ultralow detection limits	ML-Cu_2_O@Cu-MOF composite nanozyme	Electrochemical (EIS, DPV)	[89]
	*Acinetobacter baumannii*	Fluorescence amplification	Magnetic enrichment; rapid detection; high recovery efficiency	Zr-mMOF magnetic + fluorescein-loaded MOF aptasensing platform	Fluorescence	[90]
	*A. baumannii, Pseudomonas aeruginosa*	Peroxidase-like	Dual-mode detection; good performance in complex fluids	MOF–COF composite with boric acid and DNA aptamer scaffold	Fluorescence/Colorimetric	[91]
	Chlorpyrifos (pesticide, microbial sensor)	Cascade catalysis (ROS generation)	Signal amplification via in situ H_2_O_2_ generation	MOF-818@PMOF(Fe) nanozyme	Chemiluminescence/Colorimetric	[92]
	Live microbial cells (H_2_O_2_ secretion)	Peroxidase-like	Real-time metabolic monitoring; high stability	CNT/MOF composite on 3D gradient porous fiber membrane	Electrochemical	[93]
	—	Peroxidase-like	Wearable sensing; minimally invasive; colorimetric detection	DNAzyme@MOF composite in hydrogel microneedle tip	Colorimetric	[94]
	—	Peroxidase-like	Enhanced recyclability and electron transfer	Fe_3_O_4_@Au/MOF dual nanoparticle composite	Electrochemical	[95]
	—	Peroxidase-like	Responsive microenvironment; enhanced catalytic efficiency	PDM grafted UiO-66-NH_2_ polymer hybrid	Colorimetric	[96]

**Table 3:** Summary of signal transduction strategies in MOF nanozyme-based microbial biosensing.

Detection Mode	MOF Nanozyme Used	Target Analyte	Limit of Detection (LOD)	Advantage	Limitations	Ref.
Colorimetric	Cu-MOF@AF film	H_2_S, NH_3_ (spoilage gases)	-	Visible signal, pH/TVB-N/TVC correlation, field-deployable	Semi-quantitative, limited to volatile markers	[101]
Colorimetric	Aptamer-functionalized Cu-MOF	*Staphylococcus aureus*	-	High selectivity, magnetic separation	May require aptamer regeneration	[102]
Colorimetric	SapYZUs8@Cu-MOF	Viable *Staphylococcus aureus*	1.09 × 10^2^ CFU/mL	High specificity, effective in pork and milk	Target-specific, may not detect non-viable cells	[103]
Colorimetric + Electrochemical	CP@MOF (CuO_2_ nanodots in MOF)	*V. parahaemolyticus*	-	Dual mode improves accuracy (88.7%)	Complex setup	[104]
Colorimetric	MOF@MOF (Cu-MOF-808 + Fe-porphyrin MOFs)	MRSA, *Pseudomonas aeruginosa*	5 CFU/mL (MRSA), 2 CFU/mL (*Pseudomonas aeruginosa* )	Self-cascade amplifies signal, aptamer specificity	Complex synthesis	[105]
Colorimetric (Multichannel)	2D Ni–Co bimetallic MOF	Multiple microbes	-	Label-free, multichannel readout, 30 min detection	Qualitative/semi-quantitative	[106]
Colorimetric	MVCM@β-CD (2D Co-MOF)	m-Aminophenol (proxy for microbial metabolites)	0.16 μM	High catalytic efficiency, tunable surface	Not yet applied directly to microbes	[107]
Electrochemical + Colorimetric	GATC (Cu-ZIF + Au-TA + G-quadruplex/hemin aptamer)	MRSA	-	Therapeutic + diagnostic, dual-mode	Potential toxicity, multi-step synthesis	[110]
Electrochemical + Colorimetric	Gr/FeCu-NZs (Graphene + FeCu-MOF-on-MOF)	H_2_O_2_ (microbial oxidative marker)	0.06 μM	Synergistic conductivity, broad range (0.1–3800 μM)	Requires precise material tuning	[111]
Electrochemical	Ag/2D Zn-MOF	H_2_O_2_ (cellular secretion)	1.67 μM	Real time, broad range (5 μM–70 mM)	Selectivity could be improved	[112]
Fluorescence-Based	Ce-MOF	Biofilm matrix (eDNA) + H_2_O_2_	-	Therapeutic + detection, biofilm disruption	No quantification of microbial load	[117]
Fluorescence + Colorimetric (Ratiometric)	Hemin@MOF (blue-fluorescent MOF + hemin)	Tetracycline (residue)	27.2 nM (colorimetric), 4.1 nM (fluorescence)	Dual-readout, high selectivity	May be limited to specific antibiotic residues	[118]

**Table 4:** Comparative performances of nanozyme platforms for biosensing applications.

Nanozyme Type	Biomedical Application	Environmental Application	Ref.
MOF-based	Zr@ICG-NH_2_@HPW/OVA MOF nanozyme for *Pseudomonas aeruginosa* detection through GSH-depletion and photodynamic/photothermal therapy	Ce-FMA MOF nanozyme for colorimetric detection of *Escherichia coli* in water via peroxidase-like activity	[86,93]
Carbon-based	PrGO/Fe-N-C nanozyme integrated with aptamer for colorimetric detection of *Salmonella* typhimurium	3D-rGO@Au–Pt hybrid used in colorimetric paper sensor for *Salmonella* enteritidis detection in contaminated milk	[120,122]
Metal oxide-based	MnFe_2_O_4_@SiO_2_ NPs with peroxidase-like activity for sensitive detection of *Staphylococcus aureus* in infected wounds	Fe_3_O_4_ NPs in lateral flow immunoassay for *Escherichia coli* O157:H7 detection in water and food samples	[106,110]
Noble metal-based	PtCo@Au NPs integrated with aptasensor for electrochemical detection of *Salmonella* typhimurium	Au@Pt NPs for electrochemical impedance spectroscopy detection of Listeria monocytogenes in food	[128,131]
Transition metal-based	CoFe-LDH nanozyme modified electrode for electrochemical detection of Listeria monocytogenes	CuFe_2_O_4_ nanozyme-based immunosensor for *Salmonella* enteritidis detection in milk	[132,133]
Metal sulfide-based	CuS@BSA nanocluster with peroxidase-like activity for colorimetric detection of *Staphylococcus aureus*	CdS QDs@MOF hybrid for visible-light-induced photocatalytic detection of *Escherichia coli* in water	[134,135]
Hybrid nanozymes	Ag/Cu-TCPP MOF nanozyme for fluorescence and colorimetric dual-mode detection of Helicobacter pylori	Au-Pt@CeO_2_-rGO hybrid nanozyme for ultrasensitive *Escherichia coli* O157:H7 detection in wastewater	[95,126]
Polymer-based	Chitosan–Cu nanozyme for selective detection of *Escherichia coli* and *Salmonella* via smartphone-integrated colorimetry	Molecularly imprinted polymer-coated Fe_3_O_4_ nanozymes for target-specific bacterial detection in water	[136,137]
2D material-based	MoS_2_–CeO_2_ heterostructure for fluorescence biosensing of *Pseudomonas aeruginosa* via GSH-level detection	Graphene oxide/Au nanozyme composite for colorimetric detection of *Escherichia coli* in environmental water	[138,139]

**Table 5:** Summary of key challenges, implications, and proposed solutions for MOF-based nanozyme microbial biosensors.

Challenges	Implications	Proposed Solutions	Refs.
Stability and Reusability	Structural degradation and loss of catalytic activity after repeated use or harsh conditions.	Enhance structural robustness and catalytic longevity via ligand engineering, post-synthetic modifications, composites with graphene or polymers.	[139,155]
Selectivity and Specificity	Limited ability to precisely identify microbial strains in complex biological matrices.	Integrate selective biorecognition elements (aptamers, antibodies, molecularly imprinted polymers); exploit MOFs’ tunable structures for PET, RET, structural transformations.	[156,157]
Scalability and Cost	High production costs, batch inconsistencies, and difficulty in large-scale manufacturing.	Develop green, low-cost, scalable synthesis methods ensuring reproducibility and sustainability.	[158]
Smart System Integration	Technical challenges in interfacing MOFs with electronics, data processing, and communication units.	Innovate interdisciplinary solutions to integrate MOFs with AI, IoT, and smart responsive materials for adaptive biosensors.	[159]
Biosafety and Regulatory Approval	Potential cytotoxicity and environmental impact; lack of standardized testing and clear guidelines.	Conduct comprehensive toxicity and biocompatibility assessments; establish standardized protocols and regulatory frameworks.	[160,161,162]

## Abbreviations

ABTS	2,2′-azino-bis-(3-ethylbenzothiazoline-6-sulfonic acid)
AgNP	Silver nanoparticle
AuNP	Gold nanoparticle
bPEI-g-PEG	Poly(ethylene imine)-graft-poly(ethylene glycol)
CAT	Catalase
CF	Carbon microfiber
CL	Chemiluminescence
CNT	Carbon nanotube
DAP	Diaminophenazine
DHBDC^2−^	2,5-dihydroxyterephthalic acid
ECL	Electrochemiluminescence
FQ	Fluoroquinolone antibiotics
GDY-CNT	Graphdiyne/carbon nanotube
Gox	Glucose oxidase
IFE	Inner filter effect
LOD	Limit of detection
Lox	Lactate oxidase
MN	Microneedle
MOF	Metal–organic framework
NIR	Near-infrared
NP	Nanoparticle
OD	Oxidase
OMV	Outer membrane vesicle
OPD	o-phenylenediamine
PAA	Poly(acrylic acid)
PPase	Pyrophosphatase
PPi	Pyrophosphate
POD	Peroxidase
PtNP	Platinum nanoparticle
ROS	Reactive oxygen species
SOD	Superoxide dismutase
TMB	3,3′,5,5′-tetramethylbenzidine
UA	Uric acid
ZIF	Zeolitic imidazolate framework
